# Species and genus level resolution analysis of gut microbiota in *Clostridium difficile* patients following fecal microbiota transplantation

**DOI:** 10.1186/2049-2618-2-13

**Published:** 2014-04-21

**Authors:** Vijay Shankar, Matthew J Hamilton, Alexander Khoruts, Amanda Kilburn, Tatsuya Unno, Oleg Paliy, Michael J Sadowsky

**Affiliations:** 1 Department of Biochemistry and Molecular Biology, Boonshoft School of Medicine, Wright State University, 3640 Col. Glenn Hwy, Dayton, OH 45435, USA; 2 Department of Soil, Water, and Climate, Biotechnology Institute, and Microbial Plant and Genomics Institute, University of Minnesota, 140 Gortner Lab, 1479 Gortner Avenue, St. Paul, MN 55108, USA; 3 Division of Gastroenterology; Department of Medicine, Center for Immunology, University of Minnesota, Minneapolis, MN USA

**Keywords:** Microbiota, Microflora, *Clostridium difficile*, Fecal microbiota transplantation, Microbiota Array

## Abstract

**Background:**

*Clostridium difficile* is an opportunistic human intestinal pathogen, and *C. difficile* infection (CDI) is one of the main causes of antibiotic-induced diarrhea and colitis. One successful approach to combat CDI, particularly recurrent form of CDI, is through transplantation of fecal microbiota from a healthy donor to the infected patient. In this study we investigated the distal gut microbial communities of three CDI patients before and after fecal microbiota transplantation, and we compared these communities to the composition of the donor’s fecal microbiota. We utilized phylogenetic Microbiota Array, high-throughput Illumina sequencing, and fluorescent *in situ* hybridization to profile microbiota composition down to the genus and species level resolution.

**Results:**

The original patients’ microbiota had low diversity, was dominated by members of Gammaproteobacteria and Bacilli, and had low numbers of Clostridia and Bacteroidia. At the genus level, fecal samples of CDI patients were rich in members of the *Lactobacillus*, *Streptococcus*, and *Enterobacter* genera. In comparison, the donor community was dominated by Clostridia and had significantly higher diversity and evenness. The patients’ distal gut communities were completely transformed within 3 days following fecal transplantation, and these communities remained stable in each patient for at least 4 months. Despite compositional differences among recipients’ pre-treatment gut microbiota, the transplanted gut communities were highly similar among recipients post-transplantation, were indistinguishable from that of the donor, and were rich in members of *Blautia*, *Coprococcus*, and *Faecalibacterium*. In each case, the gut microbiota restoration led to a complete patient recovery and symptom alleviation.

**Conclusion:**

We conclude that *C. difficile* infection can be successfully treated by fecal microbiota transplantation and that this leads to stable transformation of the distal gut microbial community from the one abundant in aerotolerant species to that dominated by members of the Clostridia.

## Background

The use of antibiotics in modern medicine has led to a significant inhibition and in some cases complete eradication of many infectious agents that threaten human population [1]. However, the recent spread of broad-spectrum antibiotic use is also linked to an increase in the incidence of antibiotic-associated intestinal disease. Many of these incidents are caused by *Clostridium difficile*, an opportunistic human intestinal pathogen from class Clostridia. *Clostridium difficile* infection (CDI) is known to have a range of manifestations, from mild diarrhea to fulminant colitis, toxic megacolon, and death. The disease often manifests itself after treatment with antibiotics and the associated loss of resident microbiota in the intestine. Resistance of *C. difficile* to many classes of antibiotics and its ability to form spores allows this bacterium to survive antibiotic administration better than many commensal species. The decrease in commensals in the gut creates conditions favorable for a subsequent overgrowth of this opportunistic pathogen [2]. *C. difficile* spores are often acquired nosocomially, and as a result, a high incidence of CDI is seen among hospitalized patients, in the outpatient community, and among nursing home residents [3,4]. Other reservoirs of CDI can also exist according to a recent report [5].

Because of the resistance of *C. difficile* spores to antibiotics, it is challenging to cure CDI with antibiotic administration. Among alternative strategies, fecal microbiota transplantation (FMT) is gaining a wider acceptance as treatment for recurrent CDI. In this technique, fecal microbiota obtained from a healthy donor is processed, standardized, and subsequently transplanted into patients suffering from the recurrent *C. difficile* infection. FMT is highly successful (>90% success rate) and CDI symptoms often resolve within days of the transplantation procedure [6,7]. Recent studies from our group showed that the eradication of the disease symptoms is accompanied by a dramatic shift in the microbial community as examined by TRFLP and gene sequencing analyses [8,9]. However, these methods were limited in the achieved taxonomic resolution and the ability to directly quantify microbiota members, and thus they could not reveal detailed microbiota composition before and after FMT treatment.

In this study we used a human intestinal microbiota-specific phylogenetic Microbiota Array [10-13] to measure phylotype- and genus-level changes in gut microbiota of three CDI patients who underwent an FMT procedure. Microbiota Arrays contain probes targeting full-length 16S rRNA genes of 775 human microbiota phylotypes and allows direct comparison of taxon abundances between samples [10]. The microarray data were corroborated with Illumina high-throughput sequencing and fluorescent *in situ* hybridization.

## Methods

### Patients

All patients suffered from multiple recurrent *C. difficile* infection (CDI) refractory to clearance by standard antibiotic therapies, as defined previously [14]. The study of their fecal microbiota before and after FMT was approved by the University of Minnesota Institutional Review Board and all patients provided informed consent to participate in this study.

### FMT procedure

The FMT was performed using a standardized preparation of concentrated fecal microbiota as previously described [14]. Criteria for the selection of donor were described in detail previously [9,14]. The same donor was used for all recipients, but individual donations were collected on different days. Briefly, 50 g of fecal material were mixed with 250 mL of sterile phosphate buffered saline (PBS). The feces were blended, sieved, and the resulting suspension was centrifuged and washed in PBS. Patient 1 received freshly prepared material, while frozen lots were used for patients 2 and 3. We previously demonstrated FMT success with frozen fecal microbial suspension to be comparable to that obtained with a fresh preparation [14]. The patients were treated with 125 mg vancomycin, four times daily by mouth, until 2 days prior to the procedure. The day before the procedure, patients received a split dosage polyethylene glycol-based purgative (GoLYTELY®) to remove residual antibiotic and fecal material. FMT was performed via colonoscopy as previously described [14].

### Sample collection

**Figure 1:**
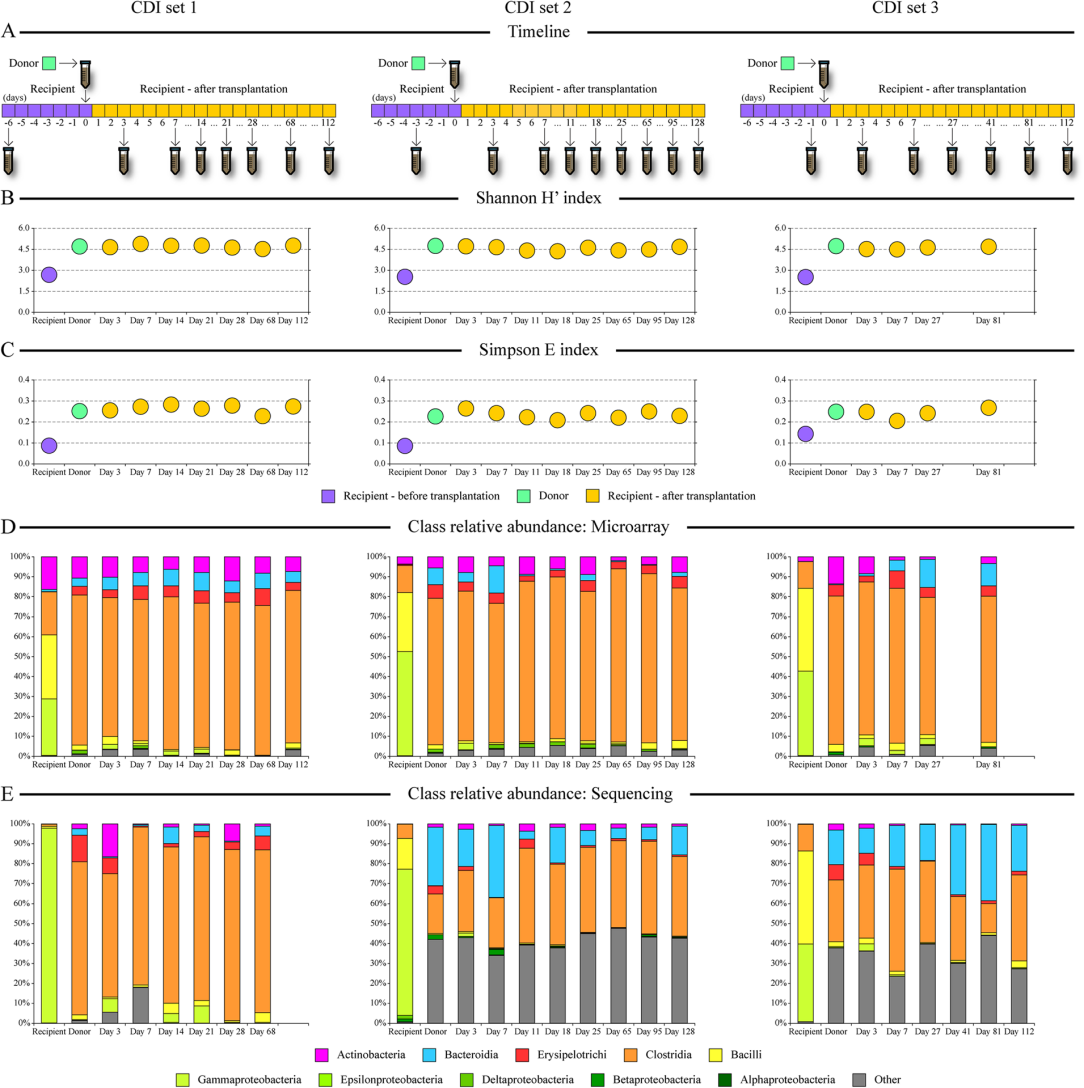
**Changes in microbiota diversity and composition following fecal transplantation in CDI patients.** Microbiota communities were profiled from three CDI patients, healthy donor, and from each patient over a 4-month period following fecal transplantation. Samples were collected periodically as shown in **(A)**. Community diversity and evenness were assessed by calculating the Shannon H’ (diversity, **B**) and Simpson E (evenness, **C**) indices based on microarray phylotype abundance data. Community structure in each sample is shown at class level in **(D)** (distribution is based on microarray data) and **(E)** (distribution is based on sequencing data). Missing data represent samples that had lower amount of fecal material available; thus not all analyses could be carried out for these samples.

Patient fecal samples were collected at home by the patients and stored frozen at approximately -20°C. The first sample for each patient was collected while the patient was receiving oral vancomycin (125 mg, four times per day) during the period up to the FMT. Samples were transferred to the laboratory within 1 week of collection and stored at -80°C until used. Donor samples for DNA extraction were collected during processing of material for FMT, and stored frozen at -80°C until used. A timeline showing sample collection for the three patients involved in this study is shown in Figure 1A.

### Isolation of genomic DNA and hybridization to Microbiota Array

Total genomic DNA (gDNA) was isolated from fecal material using ZR Fecal DNA Isolation kit (Zymo Research Corporation, procedure incorporates bead beating) according to manufacturer’s protocol. The full length bacterial 16S rRNA gene was amplified from genomic DNA with degenerate primers Bact-27Fv4 (5′-AGRGTTYGATYMTGGCTCAG-3′) and Univ-1492Rv1 (5′-GGHTACCTTGTTACGACTT-3′) [10,15]. Four separate PCR reactions were pooled together, fragmented, and then hybridized to Microbiota Array. Microbiota Array is based on Affymetrix, Inc., platform and contains sets of phylogenetic 16S rRNA gene probes (25-mer probes, 5 to 11 probe pairs in each set, each probe pair consists of perfect match and mismatch probes, each set interrogates a separate phylotype) allowing detection and enumeration of 775 bacterial phylotypes of human intestinal microbiota [10]. Microarray hybridization, washing, and scanning were carried out as described previously [12,16].

### Microarray data analysis

Raw microarray data were analyzed using previously developed pipeline [12]. Specifically, to obtain phylotype detection calls, the raw data were processed with standard MAS5 detection algorithm (Affymetrix, Inc.) using α1 = 0.03 and α2 = 0.05 parameter values. MAS5 algorithm is based on the Wilcoxon’s rank test. To obtain hybridization signal estimates for each phylotype, raw data were first normalized in CARMAweb utilizing MAS5-VSN-MAS5-MedianPolish procedure as we did previously [10]. The normalized phylotype signal values were adjusted for estimated cross-hybridization rate and unequal 16S rRNA gene copy number as described [12]. To assess our ability to separate samples based on their microbial phylotype composition, principal components (PCA) and phylogenetic principal coordinates (PCoA) analyses were used [13]. PCA, alpha diversity calculations, and permutation analyses were performed in Matlab (The Mathworks, Inc.) by employing custom written scripts. PCoA analyses were carried out on the Fast UniFrac web server [17]. Separation of genera into clusters according to genus abundances among samples was based on the K-means clustering method with manual curation to separate genera displaying patient-specific patterns of abundance changes.

### High-throughput amplicon sequencing and data analysis

DNA extractions were done as previously described [9] using MOBIO PowerSoil DNA extraction kits (MOBIO, Carlsbad, CA, USA), according to the manufacturer’s instructions. Fecal DNA samples were used as template in PCR amplification reactions of the V6 hypervariable region of the 16S rRNA gene. All PCR reactions used 25 ng of fecal DNA as template and were performed in triplicates.

DNA and amplicon preparation for high-throughput sequencing were carried out as described [9]. The samples were sequenced using Illumina Hiseq 2000 sequencer following the manufacturer’s protocols (Illumina, Hayward, CA, USA). Paired-end sequences were generated (100 nt read length) with one to three pooled samples per lane following Illumina multiplexing protocols. Paired ends were merged using a custom C script [9] by employing a minimal overlap of 25 nucleotides with 98% identity. Sequence data were processed and analyzed using the MOTHUR program [18]. Merged sequences were binned into individual sample sets according to the six nucleotide barcode sequences. The list of barcodes used and primers is provided in Additional file 1: Table S1. Sequence reads containing ambiguous bases, homopolymers larger than seven nucleotides, more than one mismatch in the primer sequence, or an average per base quality score below 25 were removed. Primer and barcode sequences were trimmed from the sequence reads prior to analysis. Sequences that only appeared once in the total set were assumed to be a result of sequencing error and were removed from the analysis. Sequences that were flagged as likely chimeras using the UCHIME algorithm were also removed from the analysis. Sequences were clustered into OTUs using the furthest neighbor algorithm with a 90% cutoff [19]. Taxonomic assignment was done using the Bayesian method with a 100 iteration bootstrap algorithm and a probability cutoff of 0.60 [9]. Summary of the obtained reads and OTU assignments are provided in the Additional file 2: Table S2. The complete sequence dataset is available in the SRA archive under bioproject number PRJNA238486 and metadata are presented in Table S6.

### Fluorescent *in situ* hybridization (FISH)

FISH was carried out as we did previously [11]. Clostridia, Bacteroidia, and Proteobacteria were visualized using FITC-labeled probes Clept1240 (5′-GTTTTRTCAACGGCAGTC-3′) + Erec482 (5′-GCTTCTTAGTCARGTACCG-3′), Bac303 (5′-CCAATGTGGGGGACCTT-3′), and Prot612 (5′-TTCCCVGGTTRAGCCCKGG-3′), respectively [11]. Following a previously optimized sample preparation protocol [11], cells were visualized under Nikon TE2000-S fluorescent microscope. In order to conduct valid comparisons between FISH and microarray results, microarray data were adjusted to account for the inability of FISH probes to detect certain genera of Clostridia and Bacteroidia (see [11] for details).

## Results

### Outcomes of the FMT procedure

We profiled intestinal microbiota in fecal samples collected from (1) three CDI patients before the FMT procedure, from (2) healthy donor, and from (3) each CDI patient over a period of 4 months following the FMT procedure. Samples were collected from each CDI patient a few days before the transplantation, on days 3 and 7 after transplantation, and then periodically with gradually increasing time periods between sample collections based on patients’ availability and ability to provide a fresh stool sample (Figure 1A). Clinically, all three CDI patients had reduced diarrheal symptoms within several days after the FMT. All patients were documented to have Undetectable *C. difficile* toxin B at 2 months by qPCR. Patients 1 and 3 had a firm bowel movement on day 3 and remained free of *C. difficile* infection for 2.5 and 2.0 years, respectively. Patient 2 had underlying ulcerative colitis, which decreased from severe pancolitis to moderate disease as evidenced by endoscopic and histological criteria 1 month after FMT. Clinically, bowel movement frequency decreased from 10-12/day to 4/day, and rectal bleeding and tenesmus resolved. Colonoscopy at 1 year showed endoscopically moderate disease in the distal colon, but near normal appearance in the proximal colon. This patient remained free of *C. difficile* infection for 1.5 years until treated with antibiotics for a urinary tract infection, at which time he experienced a re-infection with *C. difficile*.

### Changes in distal gut microbial community composition in CDI patients following fecal transplantation

The microarray phylotype abundance data were used to assess the differences in the intestinal microbial communities among all samples. The ecological organization of the communities was calculated using the Shannon’s diversity (Figure 1B) and the Simpson’s evenness (Figure 1C) indices. Overall, the gut microbiota in CDI patients had low diversity and evenness in all three patients prior to FMT (Shannon’s H’ = 2.58 ± 0.09 and Simpson E = 0.11 ± 0.03). The communities were dominated by relatively few members with high abundance, and the overall number of species in the community was low. In contrast, the microbiota from the healthy donor showed statistically significantly higher diversity and evenness (H’ = 4.73 ± 0.02 and E = 0.24 ± 0.01; α < 10^−5^ and α = 0.001, respectively, based on a one-tail *T*-test). After-transplantation samples from day 3 onward had increased diversity and evenness similar to those of the donor microbiota (H’ = 4.61 ± 0.14 and E = 0.25 ± 0.02; α = 0.18 and α = 0.74 based on a two-tail *T*-test of donor and after-FMT sample comparison). Similar effects of microbiota transplantation (delivered as duodenal infusion) on gut microbial diversity in CDI patients were also recently observed by van Nood and co-workers [7]. The overall community structure remained remarkably stable through the 4-month sampling period (average Spearman correlation of microarray-determined genus abundances between consecutive time points was 0.87 for after transplantation samples) and at the end of the observation both community diversity and evenness remained similar to that of the donor (H’ = 4.71 ± 0.05 and E = 0.26 ± 0.02).

At the class level, microarray results revealed considerable changes between the microbiota profiles before and after transplantation (Figure 1D and Additional file 3: Table S3). In all three CDI patients, their fecal samples before transplantation contained relatively high abundance of organisms belonging to classes Gammaproteobacteria (40.9% cumulative abundance on average) and Bacilli (34.5% on average). Donor samples were dominated by the classes Clostridia (74.5%), Actinobacteria (10.0%), Erysipelotrichi (5.6%), and Bacteroidia (4.3%). Concordantly, the recipients’ after-transplantation samples showed increases in Bacteroidia (from 0.5% to 6.1% on average) and Clostridia (from 16.1% to 75.5%), with Proteobacteria (2.1%) and Bacilli (2.0%) in low abundance. The microarray class level data also indicated an overall stability of the microbial community structure in the after-transplantation distal gut over the entire follow-up period (Figure 1D). The dramatic shifts in the abundances of microbial classes following FMT were also evidenced by Illumina sequencing (Figure 1E), although the sequencing results showed somewhat greater community variability among the after-transplantation samples. The observed greater robustness of microarray data is likely explained by the ability of microarray to measure presence and abundance of each interrogated phylotype in each sample [12]. Thus, the microarray results are not dependent on the sequencing depth and are less sensitive to any 16S rRNA gene PCR amplification biases.

**Figure 2:**
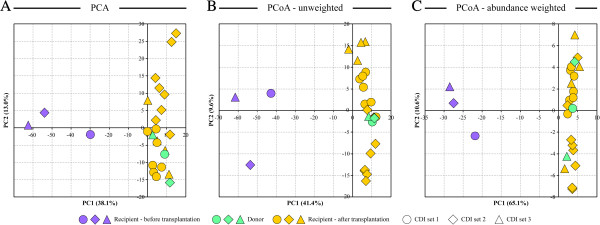
**Separation of samples based on ordination multivariate analysis of microarray phylotype abundance data.** Principal components analysis (PCA, **A**) and unweighted (separation is based on phylotype presence, **B**) and weighted (separation is based on phylotype presence and abundance, **C**) principal coordinates analysis (PCoA) show separation of recipient samples before transplantation from both donor and recipient samples obtained after transplantation. Percent of dataset variability explained by each principal component/coordinate is shown in brackets in axis titles.

Utilizing phylotype abundance data obtained with Microbiota Array, sampled microbial communities were also analyzed with high-dimensionality reducing principal components analysis (PCA) and phylogenetic principal coordinates analysis (PCoA) ordination approaches [13]. All ordination analyses showed a clear separation of recipients’ before-transplantation samples from those of the donor and the patients’ after-transplantation samples (Figure 2). Consistent with our analysis as described above, there was a considerable difference in the community phylotype structure between the CDI and donor samples, and this difference was the largest determinant of dataset variability, because the donor and CDI samples were separated along the principal component/coordinate 1 axis representing the highest data variability [20]. A significant degree of variability was seen among the recipients before transplantation especially in the phylotype presence (Figure 2B), indicating that pre-transplantation gut microbial communities were unique to each profiled CDI patient. The after-transplantation samples for all three recipients clustered together with donor samples, demonstrating that compositional individuality of pre-treatment gut microbiota had little influence on the post-FMT microbial community structure. Post-FMT distal gut microbiota structure was thus determined by donor microbiota community.

### Visualization of fecal microbiota with FISH

**Figure 3:**
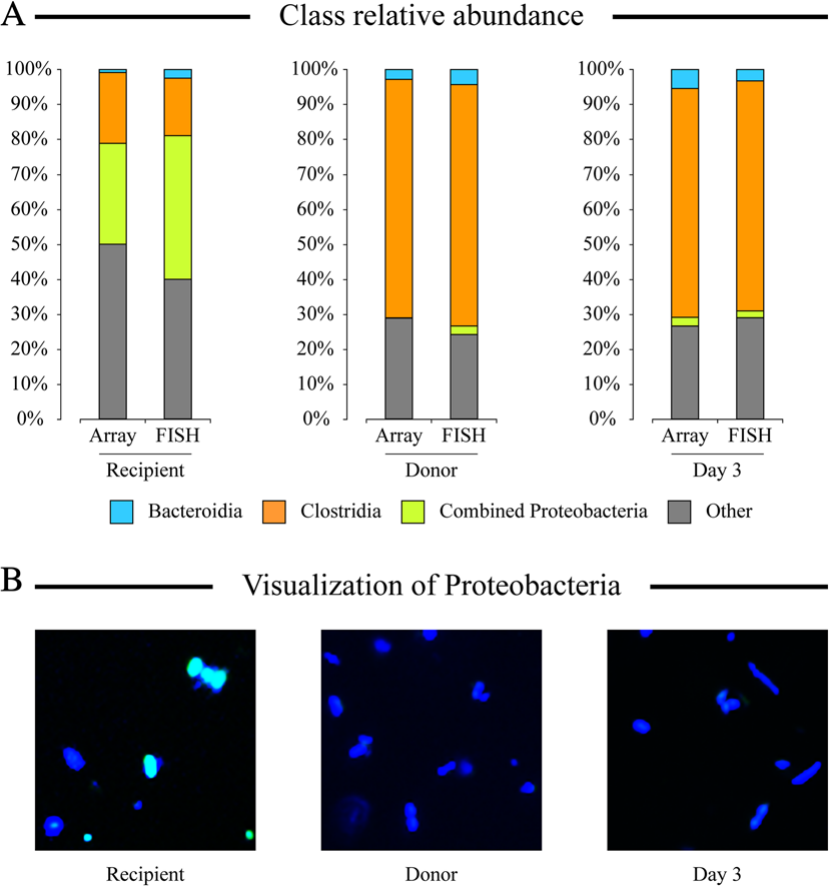
**Quantification of bacterial abundances using fluorescent*in situ*hybridization.** Select fecal samples from CDI set 1 were profiled using class specific DNA probes as shown. **(A)** A comparison of class relative abundances measured by Microbiota Array and by FISH. **(B)** Representative captured image from each sample visualized with Proteobacteria fluorescent probe (green color) and DAPI DNA stain (blue color).

Because fluorescent *in situ* hybridization allows cell quantitation through direct visualization, it is a good choice for validation of results obtained through DNA-based techniques such as phylogenetic microarrays or high-throughput sequencing. With that goal, we utilized FISH to visualize and quantify Bacteroidia, Clostridia, and Proteobacteria in three fecal samples from CDI set 1: recipients before transplantation, donor, and samples obtained from the same patient 3 days after transplantation. As shown in Figure 3A, the taxon abundances obtained from FISH quantitation matched well those from the microarray results, with only somewhat higher estimate of Proteobacteria in the recipient pre-transplantation sample based on FISH (28.8% and 41.0% based on array and FISH calculations, respectively, note that microarray data were adjusted to match each FISH probe inability to detect some members of each class/phylum). A representative image from each sample examined by FISH using a Proteobacteria probe and a generic DNA stain (DAPI) are shown in Figure 3B; Proteobacteria cells were largely detected only on a slide with initial recipient microbiota but not in the other two samples.

### Specific genera are responsible for observed microbiota differences among samples

**Figure 4:**
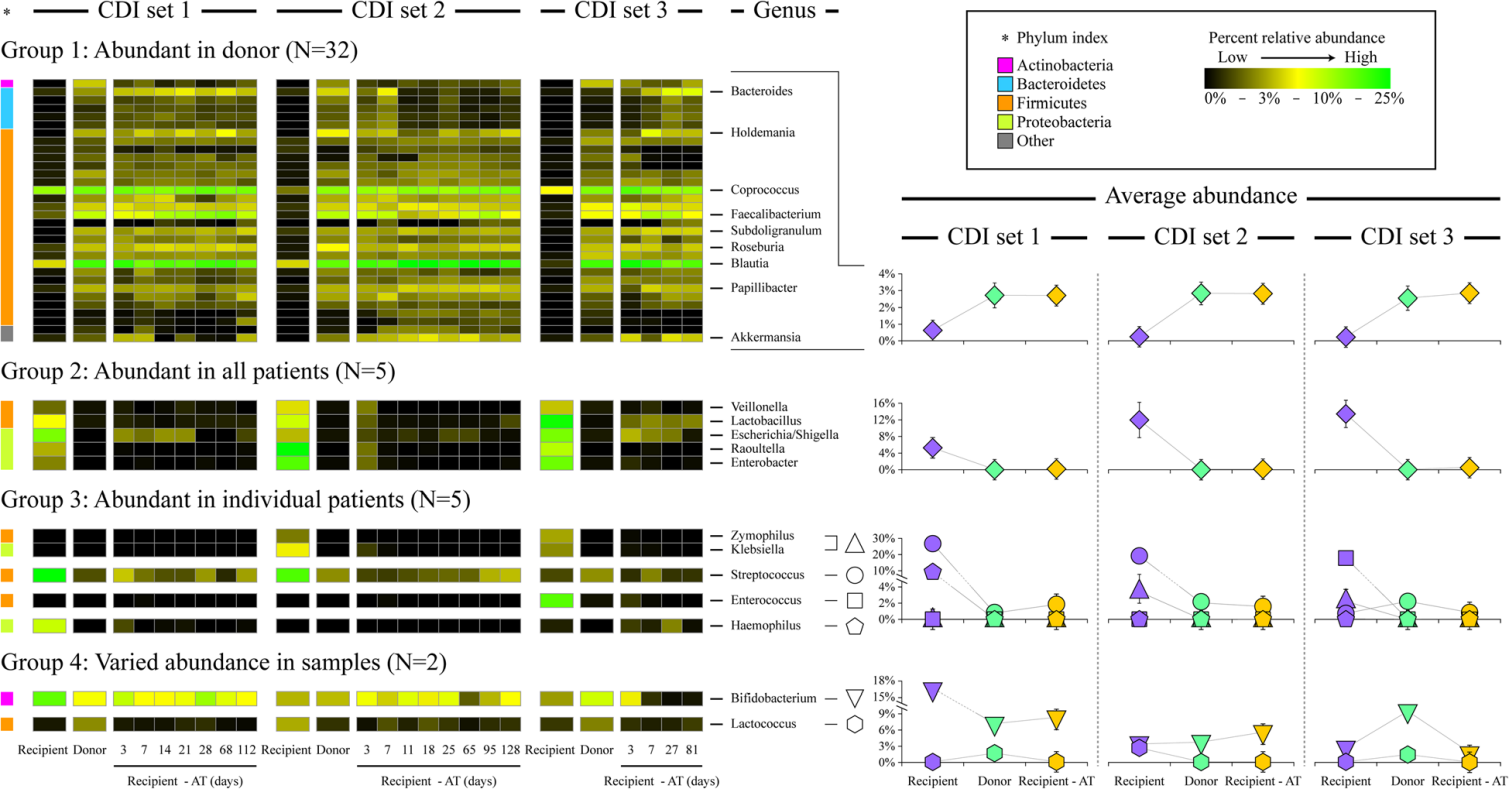
**Relative abundances of bacterial genera in all samples as measured by Microbiota Array.** All genera were distributed into groups based on the analysis of genus abundances across samples. A group of genera that were not detected consistently in samples or were detected at very low level is not shown (N = 86). A heat-map of genus abundances is shown on the left-hand side of the image. Gradient color scheme and phylum designation are displayed in the legend. An average abundance of all genera in each type of samples is shown for groups 1 and 2 on line graphs on the right-hand side. For groups 3 and 4, the line graphs display individual genus values. The abundances of after-treatment time points were averaged together. Where possible, error bars were calculated to represent standard error of the mean.

Because Microbiota Arrays contain probes to individual microbial phylotypes, its use allowed a quantitative assessment of phylotype and genus level abundances in all samples. We thus sought to compare genus abundances among samples and to define groups of genera that displayed similar patterns across the sample set. Genera were distributed into five groups based on their abundance values among samples (Figure 4, Additional file 4: Figure S1, and Additional file 5: Table S4). Group 1 comprised genera that were present at high abundance in the donor and after-transplantation samples but which were not highly abundant in CDI patients’ samples prior to FMT procedure. Notable members included a number of genera from the classes Clostridia and Bacteroidia, such as *Bacteroides*, *Blautia*, *Coprococcus*, *Faecalibacterium*, *Papillibacter*, and *Roseburia.* These taxa are known to comprise a substantial portion of human colonic microbiota and to play important roles in the energy metabolism and commensal host-microbial interactions [21]. Among these, only *Blautia* and *Coprococcus* were also somewhat abundant in the patient before-transplantation samples (Figure 4, group 1).

Group 2 consisted of five facultatively anaerobic and/or aerotolerant genera *Enterobacter*, *Escherichia*, *Lactobacillus*, *Raoultella*, and *Veillonella*; these were present at high abundance in all three before-transplantation samples, but not in donor samples. The levels of these genera were similarly low in the fecal samples of CDI patients after FMT. Genera that we assigned to group 3 were abundant in some but not all before-transplantation samples, while their levels in donor and after-transplantation samples were similarly low. These included *Streptococcus* (abundant in patients 1 and 2), *Zymophilus* and *Klebsiella* (abundant in patients 2 and 3), *Haemophilus* (only found in patient 1), and *Enterococcus* (only in patient 3). Two genera - *Bifidobacterium* and *Lactococcus* - did not show consistent patterns across donor, before-, and after-transplantation samples and were thus assigned to group 4.

Group 5 comprised genera that were not consistently detected in samples or detected with very low abundance (N = 86, see Additional file 4: Figure S1). Similar to the findings shown in Figure 1, genera present in high abundance in the donor samples were also highly abundant in the after-transplantation samples. Conversely, genera present in high abundance in all or some of the recipient before-transplantation samples were in relatively lower abundance in both the donor and after-transplantation samples. This indicates a complete reorganization of the gut microbiota in CDI patients following FMT procedure.

### Species level changes following FMT

The distribution of phylotypes and species generally followed the pattern observed for genera, with species of Clostridia and Bacteroidia generally scarce or undetected in pre-FMT samples but abundant in donor and after-FMT samples. While microarray allows enumeration of 775 different microbial phylotypes, only about 65 of them are assigned to known microbial species [10]. Among these, *Bacteroides fragilis*, *B. ovatus*, and *B. uniformis* were not present above 0.1% abundance level in any of the original patient samples, but increased to an average cumulative abundance of 1.9% after transplantation. Similar increases in after-FMT samples were observed for *Faecalibacterium prausnitzii*, *Clostridium bartlettii*, *Dorea longicatena*, *Holdemania filiformis*, *Roseburia intestinalis*, and *Ruminococcus obeum*. Interestingly, even though the microarrays contained a probe-set for *Clostridium difficile*, this species was not confidently detected in any of the original CDI patients’ fecal samples, possibly because the vegetative *C. difficile* cells were largely eradicated by antibiotic treatment, whereas DNA isolation from *C. difficile* spores might not have been successful [22]. Concordantly, *C. difficile* toxin B was also not detected in these samples with clinical qPCR test. In contrast, individual patient’s pre-transplantation samples contained remarkably high abundance levels of a few other species: - *Bifidobacterium adolescentis* (12.8% of overall abundance) and *Escherichia coli* (3.5%) in patient 1, *Klebsiella pneumonia* (5.5%) and *Bifidobacterium adolescentis* (3.1%) in patient 2, and *Enterococcus faecium* (17.1%), *Lactobacillus salivarius* (6.7%), and *Escherichia coli* (3.3%) in patient 3.

## Discussion

The goal of this study was to profile phylotype-level microbiota composition in three CDI patients and to follow microbiota changes during and after FMT. While several previous reports have evaluated microbiota alterations following FMT therapy in human patients [7-9,23,24], the genus and species level changes were explored only in few studies [25]. The utilization of phylogenetic Microbiota Array allowed us to obtain quantitative measurements of different taxonomical groups in all samples, and microarrays uniquely generated genus and species level resolution of distal gut microbiota in these samples. We used Illumina high-throughput sequencing and FISH to provide additional support for our findings.

The analyses presented here show that the colon of each CDI patient was host to a severely compromised intestinal microbial community, which was significantly reduced in diversity and richness. This was likely a result of *C. difficile* proliferation as well as due to the antibiotic treatment used in an attempt to rid the disease. Ulcerative colitis, a common coexisting condition in patients with recurrent CDI [14,26], may have contributed to dysbiosis in patient 2, although typically ulcerative colitis alone does not significantly decrease microbial diversity [27]. While the healthy gut microbiota is usually dominated by Clostridia and Bacteroidia, fecal samples of the CDI patients were abundant in Gammaproteobacteria and Bacilli. Interestingly, both of these abundant taxons contain many facultative anaerobic or aerotolerant bacteria, and many are known to be abundant in the human ileum, in part due to their ability to tolerate the presence of oxygen [28-33]. Normally such species contain genes coding for proteins that allow cells to ameliorate toxic reactive oxygen species (ROS), such as hydrogen peroxide and superoxide O_2_^−^. In addition to the tolerance against molecular oxygen and its effects, these oxidative stress response genes also provide protection against ROS generated by neutrophils and macrophages and released into the gut during inflammation [34]. We found the presence of at least two of four genes coding for either catalase (decomposes hydrogen peroxide), superoxide dismutase (detoxifies superoxide), glutathione synthase (glutathione serves as antioxidant), or glutathione peroxidase (reduces hydrogen peroxide to water) in the genome sequences of human gut representatives of *Enterobacter*, *Escherichia*, *Lactobacillus*, *Veillonella*, *Klebsiella*, *Haemophilus*, *Enterococcus*, and *Streptococcus* (see Additional file 6: Table S5). These genera were found to be highly abundant in the fecal samples of CDI patients, an observation partially matched by the analysis of CDI microbiota by Anthraham and colleagues [35]. In contrast, the genomes of several prominent obligate anaerobes from the human gut such as *Faecalibacterium prausnitzii* and *Roseburia intestinalis* did not contain any of these genes.

We offer three potential explanations for the observed composition of the fecal microbiota in CDI patients. 1) Because *C. difficile* infection is associated with frequent diarrhea, it usually leads to a significantly shortened transit time in the large intestine [36], and diarrhea would result in a lower overall load of microbes in the colon [37]. This may cause significantly fewer members of the colonic communities being present in the fecal material, and as a consequence it will lead to an increase of relative abundances of small intestinal genera in feces (these can be shed in higher amounts into the colonic lumen due to faster gut transit time). 2) Because diarrhea in combination with antibiotic administration during standard CDI treatment are expected to reduce the overall counts of microbes in the colon [38], this may increase the average oxygen level in that gut region (typically less than 1% [39]). Additionally, reactive oxygen species can be released in the gut of CDI patients by macrophages and neutrophils, which are recruited into gut mucosa during CDI development [40]. Both of the described effects will create an environment selectively more advantageous for the species that are able to tolerate the presence of oxygen and ROS. 3) Antibiotic administration alone can play a role in shaping the composition of the intestinal microbial community. A standard antibiotic regimen in CDI included metronidazole followed by multiple cycles of vancomycin, and patient 2 also received rifaximin. Since metronidazole predominantly targets anaerobic bacteria [41], and vancomycin inhibits cell wall synthesis of gram-positive bacteria [42], the use of these antibiotics would be expected to reduce members of class Clostridia (most are strictly anaerobic gram-positive species) and not affect Proteobacteria (many are facultatively anaerobic gram-negative species). It is likely that a combination of the factors described impart a selective pressure on the gut microbial communities in CDI patients. This in turn likely leads to a relative decrease in the abundance of obligate anaerobic species and an increase in the presence of aerotolerant members in the distal gut and fecal matter.

In contrast to the fecal communities of CDI patients, the fecal samples of the donor were dominated by members of Clostridia, Actinobacteria, Erysipelotrichi, and Bacteroidia, which is a common composition of the distal gut microbiota in healthy humans [43]. The FMT procedure rapidly and drastically altered the intestinal microbiota communities in CDI patients, both in the taxonomy of organisms present and in their relative abundances, so that even at day 3 after FMT the recipient’s microbiota matched that of the donor. Such microbiota restructuring after FMT was also noted in our previous reports as well as in several other studies [8,9,24,44]. Genus and species level changes in CDI patients upon FMT were also reported by Shahinas and colleagues, though the success of community reorganization varied from case to case in that study [25]. In all our patients, the composition of these engrafted communities remained stable over the 4-month observation period, and the structure of the microbiota depended upon the original composition of the donor community. This microbial compositional shift was accompanied by the cessation of CDI symptoms.

## Conclusion

Several reports have indicated the overwhelming clinical efficacy of FMT [7,14,45,46]. In contrast to probiotic therapy, which introduces a limited number of micro-organisms into the intestinal tract, FMT effectively replaces the entire colonic microbiota with a healthy one in order to reestablish the lost intestinal homeostasis. The success of FMT to treat *C. difficile* infection, revealed in this and other recent studies, also opens possibilities for application of this approach to other gastrointestinal disorders. Conditions such as inflammatory bowel disease and metabolic disorders including malnutrition and obesity, where the resident gut microbiota is thought to be a key contributing factor, are possible candidates to which FMT could be applied with success [47,48].

## Abbreviations

CDI: *C. difficile* infection; FISH: Fluorescent *in situ* hybridization; FMT: Fecal microbiota transplantation; gDNA: Genomic DNA; PCA: Principal components analysis; PCoA: Principal coordinates analysis; ROS: Reactive oxygen species.

## Competing interests

The authors declare that they have no competing interests.

## Authors’ contributions

AlK, OP, and MJS have developed study concept and design; VS, MJH, AmK, and TU acquired the data; VS, MJH, and OP analyzed the data; VS, MJH, MJS, and OP wrote the manuscript. All authors read and approved the final manuscript.

## Supplementary Material

Additional file 1: Table S1 Primer sequences and barcodes used for Illumina-based sequencing. Click here for file

Additional file 2: Table S2 Cumulative number of Illumina reads and assigned OTUs per sample. Click here for file

Additional file 3: Table S3 Class level abundances among all profiled samples. Click here for file

Additional file 4: Figure S1 Expanded version of Figure 4 showing all 130 profiled microbial genera. Click here for file

Additional file 5: Table S4 Genus level abundances among all profiled samples. Click here for file

Additional file 6: Table S5 Presence of ROS detoxifying genes among select genera of human gut microbiota. Click here for file
