# Supplementary material for: Species and genus level resolution analysis of gut microbiota in Clostridium difficile patients following fecal microbiota transplantation
Source: Microbiome. 2014 Apr 21;2:13. doi: 10.1186/2049-2618-2-13 (PMC4030581; doi:10.1186/2049-2618-2-13)
Supplement: Additional file 1: Table S1 — Primer sequences and barcodes used for Illumina-based sequencing. [file 2049-2618-2-13-S1.pdf]

Species	Catalase		Glutathione synthase		Glutathione peroxidase		Superoxide dismutase	
	Gene name	***Locus tag***	***Gene name***	***Locus tag***	***Gene name***	***Locus tag***	***Gene name***	***Locus tag***
Facultatively anaerobic and aerotolerant members								
Enterobacter cancerogenus ATCC 35316	KatE	ENTCAN_05767	Gsh	ENTCAN_08918	Gpo	ENTCAN_05788	SodC	ENTCAN_05870
Enterobacter cloacae subsp. cloacae NCTC 9394	KatE	ENC_14320	Gsh	ENC_31970	Gpo	ENC_14090	Sod_Fe	ENC_00700
Enterobacter bacterium 9_2_54FAA	KatE	HMPREF0864_01758	Gsh	HMPREF0864_02725	-	-	Sod_Fe	HMPREF0864_01313
Enterococcus faecalis PC1.1	KatE	CUI_0574	-	-	-	-	SodA	CUI_0105
Enterococcus faecium TX1330	YdbD	HMPREF0352_2270	GshAB	HMPREF0352_0714	Gpo	HMPREF0352_2321	SodA	HMPREF0352_0471
Enterococcus saccharolyticus 30_1	-	-	GshAB	HMPREF9478_00294	Gpo	HMPREF9478_01187	SodA	HMPREF9478_02030
Escherichia coli SE15	KatE	ECSF_1593	Gsh	ECSF_2746	Gpo	ECSF_1571	Sod_Fe	ECSF_3769
Escherichia sp. 1_1_43	KatE	ESCG_03746	Gsh	ESCG_00328	-	-	SodC	ESCG_03668
Escherichia sp. 3_2_53FAA	KatE	ESAG_02409	Gsh	ESAG_01411	-	-	SodC	ESAG_02319
Escherichia sp. 4_1_40B	KatE	ESBG_00949	Gsh	ESBG_03010	Gpo	ESBG_00972	Sod_Fe	ESBG_01027
Haemophilus influenza 10810	KatE	HIB_10660	GshAB	HIB_00480	-	-	Sod_Fe	HIB_12460
Klebsiella oxytoca E718	KatE	A225_4143	Gsh	A225_4974	Gpo	A225_3249	Sod_Fe	A225_3300
Klebsiella sp. 1_1_55	KatE	HMPREF0485_04684	Gsh	HMPREF0485_03119	Gpo	HMPREF0485_04557	SodC	HMPREF0485_01012
Klebsiella sp. 4_1_44FAA	KatE	HMPREF1024_03537	Gsh	HMPREF1024_02527	-	-	SodC	HMPREF1024_00743
Klebsiella sp. MS 92-3	KatE	HMPREF9538_05106	Gsh	HMPREF9538_00816	Gpo	HMPREF9538_04240	Sod_Fe	HMPREF9538_02643
Lactobacillus casei BL23	-	-	GshAB	LCABL_14150	Gpo	LCABL_10060	SodA	LCABL_20710
Lactobacillus plantarum subsp. plantarum ATCC 14917	KatA	HMPREF0531_12395	GshAB	HMPREF0531_13051	Gpo	HMPREF0531_10291	-	-
Lactobacillus rhamnosus GG	-	-	Gsh	LRHM_1159	Gpo	LRHM_0814	-	-
Streptococcus anginosus 1_2_62CV	-	-	Gsh	HMPREF9459_01363	-	-	SodA	HMPREF9459_00862
Streptococcus equinus ATCC 9812	-	-	Gsh	HMPREF0819_0583	Gpo	HMPREF0819_0897	SodA	HMPREF0819_0113
Streptococcus infantarius subsp. infantarius CJ18	-	-	GshAB	Sinf_1855	Gpo	Sinf_1336	SodA	Sinf_0547
Veillonella sp. 3_1_44	KatE	HMPREF0873_00907	-	-	Gpo	HMPREF0873_00936	SodA	HMPREF0873_00927
Veillonella sp. 6_1_27	KatE	HMPREF0874_00962	-	-	Gpo	HMPREF0874_00994	SodA	HMPREF0874_00983
Obligate anaerobic members								
Bifidobacterium longum subsp. infantis 157F	-	-	-	-	-	-	-	-
Faecalibacterium prausnitzii M21/2	-	-	-	-	-	-	-	-
Roseburia intestinalis M50/1	-	-	-	-	-	-	-	-
Subdoligranulum variabile DSM 15176	-	-	-	-	-	-	-	-

[Information was obtained using NCBI protein database for human gut microbiota species with genomes sequenced by the Human Microbiome Project]
