# Supplementary material for: Species and genus level resolution analysis of gut microbiota in Clostridium difficile patients following fecal microbiota transplantation
Source: Microbiome. 2014 Apr 21;2:13. doi: 10.1186/2049-2618-2-13 (PMC4030581; doi:10.1186/2049-2618-2-13)
Supplement: Additional file 4: Figure S1 — Expanded version of Figure 4 showing all 130 profiled microbial genera. [file 2049-2618-2-13-S4.pdf]

Primers used in amplification

Primer designation	***Sequence***
Forward Primer 1	CNA CGC GAA GAA CC T TAN C
Forward Primer 2	CAA CGC GAA AAA CC T TAC C
Forward Primer 3	CAA CGC GCA GAA CC T TAC C
Forward Primer 4	ATA CGC GAR GAA CC T TAC C
Forward Primer 5	CTA ACC GAN GAA CC T YAC C
Reverse Primer 1	[6bp ID tag]CGA CAG CC A TGC ANC ACC T
Reverse Primer 2	[6bp ID tag]CGA CAA CC A TGC ANC ACC T
Reverse Primer 3	[6bp ID tag]CGA CGG CC A TGC ANC ACC T
Reverse Primer 4	[6bp ID tag]CGA CGA CC A TGC ANC ACC T

Barcodes used

Patient ID	***Sample ID***	***Barcode***
CDI set 1	*Recipient*	CGATGT
CDI set 1	*Donor*	ATCACG
CDI set 1	*Day 3*	TTAGGC
CDI set 1	*Day 7*	TGACCA
CDI set 1	*Day 14*	ACAGTG
CDI set 1	*Day 21*	GCCAAT
CDI set 1	*Day 28*	CAGATC
CDI set 1	*Day 68*	ACTTGA
CDI set 2	*Recipient*	TTAGGC
CDI set 2	*Donor*	ATCACG
CDI set 2	*Day 3*	TGACCA
CDI set 2	*Day 7*	ACAGTG
CDI set 2	*Day 11*	GCCAAT
CDI set 2	*Day 18*	CAGATC
CDI set 2	*Day 25*	ACTTGA
CDI set 2	*Day 65*	GATCAG
CDI set 2	*Day 95*	TAGCTT
CDI set 2	*Day 128*	GGCTAC
CDI set 3	*Recipient*	GGTTAA
CDI set 3	*Donor*	CTTGTA
CDI set 3	*Day 3*	CCAGGA
CDI set 3	*Day 7*	GGCAGT
CDI set 3	*Day 27*	AAGACT
CDI set 3	*Day 81*	TTTCCT
